# Differential gene expression of serine protease inhibitors in bovine ovarian follicle: possible involvement in follicular growth and atresia

**DOI:** 10.1186/1477-7827-9-72

**Published:** 2011-05-27

**Authors:** Ken-Go Hayashi, Koichi Ushizawa, Misa Hosoe, Toru Takahashi

**Affiliations:** 1 Reproductive Biology Research Unit, Division of Animal Science, National Institute of Agrobiological Sciences, Tsukuba 305-8602, Japan

## Abstract

**Background:**

SERPINs (serine protease inhibitors) regulate proteases involving fibrinolysis, coagulation, inflammation, cell mobility, cellular differentiation and apoptosis. This study aimed to investigate differentially expressed genes of members of the SERPIN superfamily between healthy and atretic follicles using a combination of microarray and quantitative real-time PCR (QPCR) analysis. In addition, we further determined mRNA and protein localization of identified SERPINs in estradiol (E2)-active and E2-inactive follicles by *in situ *hybridization and immunohistochemistry.

**Methods:**

We performed microarray analysis of healthy (10.7 +/- 0.7 mm) and atretic (7.8 +/- 0.2 mm) follicles using a custom-made bovine oligonucleotide microarray to screen differentially expressed genes encoding SERPIN superfamily members between groups. The expression profiles of six identified SERPIN genes were further confirmed by QPCR analysis. In addition, mRNA and protein localization of four SERPINs was investigated in E2-active and E2-inactive follicles using *in situ *hybridization and immunohistochemistry.

**Results:**

We have identified 11 SERPIN genes expressed in healthy and atretic follicles by microarray analysis. QPCR analysis confirmed that mRNA expression of four SERPINs (*SERPINA5, SERPINB6, SERPINE2 *and *SERPINF2*) was greater in healthy than in atretic follicles, while two SERPINs (*SERPINE1 *and *SERPING1*) had greater expression in atretic than in healthy follicles. *In situ *hybridization showed that *SERPINA5, SERPINB6 *and *SERPINF2 *mRNA were localized in GCs of E2-active follicles and weakly expressed in GCs of E2-inactive follicles. *SERPING1 *mRNA was localized in both GCs and the theca layer (TL) of E2-inactive follicles and a weak hybridization signal was also detected in both GCs and TL of E2-active follicles. Immunohistochemistry showed that SERPINA5, SERPINB6 and SERPINF2 were detected in GCs of E2-active and E2-inactive follicles. SERPING1 protein was localized in both GCs and the TL of E2-active and E2-inactive follicles.

**Conclusions:**

Our results demonstrate a characteristic expression of SERPIN superfamily member genes in bovine healthy and atretic follicles. The cell-type-and stage-specific expression of SERPINs may be associated with bovine follicular growth and atresia.

## Background

The final growth of bovine antral follicles shows a wave-like pattern [1,2]. In each follicular wave, usually one dominant follicle (DF) is selected from a cohort of growing follicles and continues growth while other follicles undergo atresia [3]. The DF is characterized by expression of luteinizing hormone (LH) receptor in granulosa cells (GCs) and enhanced estradiol (E_2_) production [4]. Various intrafollicular molecules, including the insulin-like growth factor family and the transforming growth factor-β family, play a crucial role in regulating DF selection and its further growth [5,6]. In addition, increasing evidence has revealed that numerous genes are regulated during bovine follicular development and ovulation through the use of global transcription profiling such as microarray analysis [7-15]. Recently, we investigated differences in global gene expression profiles between bovine healthy follicles and atretic follicles using a bovine oligonucleotide microarray [15]. In that study 76 differentially expressed genes between the follicles were identified, demonstrating that gene expression in the follicles may be closely associated with their developmental status (healthy or atretic). Also in that study, we found several genes cording for serine protease inhibitors (SERPINs), being expressed in both healthy and atretic follicles.

SERPINs comprise a huge superfamily of protease inhibitors with similar structures that undergo conformational changes in the formation of stable complexes between inhibitor and target enzymes [16]. Most SERPINs inactivate serine proteases and some cystein proteases, and they play a functional role in diverse biological processes including fibrinolysis, coagulation, inflammation, cell mobility, cellular differentiation and apoptosis [16,17]. It has been reported that expression and secretion of three SERPINs, SERPINB2, SERPINE1 and SERPINE2 change in a stage-dependent manner during bovine follicular development and in the periovulatory period [18-21]. The mRNA expression of these SERPINs in preovulatory follicles was markedly up-regulated immediately after the beginning of the LH surge, then decreased to a nadir level near the time of ovulation [18,21]. During follicular development, *SERPINE2 *mRNA levels were higher in GCs of DF compared to small follicles [19] while the follicular fluid (FF) concentration of SERPINE2 was significantly higher in non-atretic than in atretic follicles [21]. An *in vitro *study demonstrated that cultured GCs from large follicles secreted more SERPINE2 than GCs from small and medium-sized follicles [20]. All of these three SERPINs are involved in the regulation of follicular extracellular matrix (ECM) remodeling to inhibit activity of plasminogen activators (PAs) and/or plasmin.

Even though a number of SERPINs with various functions are known, the presence of other SERPINs except for the above three SERPINs have not been examined in bovine follicles. We hypothesized that temporal and cell-specific regulation of SERPIN expression could contribute to follicular development in cattle. The aim of this study was to identify differentially expressed SERPIN genes between healthy and atretic follicles using a combination of microarray analysis and quantitative real-time PCR (QPCR) analysis. Moreover, mRNA and protein localization of several identified SERPINs was further investigated in E_2_-active and E_2_-inactive follicles using *in situ *hybridization and immunohistochemistry.

## Methods

In the present study, the follicles used in experiment 1 and 2 were those previously used in our study [15]. The details of procedures for sample collection of experiment 1 and 2, RNA extraction, microarray analysis, QPCR analysis, steroid hormone determinations and *in situ *hybridization have been described in our previous report [15]. All procedures for animal experiments were carried out in accordance with guidelines approved by the Animal Ethics Committee of the National Institute of Agrobiological Sciences for the use of animals.

### Experiment 1: identification of differentially expressed SERPIN genes by microarray analysis and QPCR analysis

#### Sample collection and RNA extraction

Paired ovaries were obtained from four pregnant Japanese Black cows (day 20-27 of pregnancy) in the institute ranch less than 10 min after slaughtering. These cows were pregnant and slaughtered for another study. Follicles that have the largest diameter and second-largest diameter within the paired ovaries were dissected, snap-frozen and stored at -80°C until RNA extraction. We collected three largest and three second-largest follicles from four cows because two cows had both largest and second-largest follicles collected whereas one cow had only one largest follicle collected and another cow had only one second-largest follicle collected. As described in our previous study [15], we evaluated that the largest follicles were healthy while the second-largest follicles were atretic by differences in follicular gene expression profiles. The mean diameter of largest (healthy) and second-largest (atretic) follicles were 10.7 ± 0.7 mm and 7.8 ± 0.2 mm, respectively. Total RNA from the follicular wall (i.e., granulosa plus theca interna) was extracted from each follicle using ISOGEN (NipponGene, Tokyo, Japan) according to the manufacturer's instructions.

#### Microarray analysis and quantitative real-time RT-PCR analysis

We used a custom-made bovine oligonucleotide microarray fabricated by Agilent Technologies (Santa Clara, CA, USA). Sixty-mer nucleotides probes for customized microarray were synthesized on a glass slide. The annotated bovine oligonucleotide array represented 10263 sequences of which 4466 genes were known bovine genes including 14 SERPIN genes, 5697 unknown sequences that were possible candidates for novel bovine genes, and 100 internal references. We performed one-color microarray using five follicles (three healthy follicles and two atretic follicles) as previously described [15]. After data normalization, 3308 genes were left to use for further analysis. The relative abundance of individual genes between follicles was calculated by dividing the normalized value of the genes between each follicle. Then, we picked up SERPIN genes that showed fluorescence intensities differing by at least a 2.0-fold induction or a 0.5-fold repression between healthy and atretic follicles and defined these genes as a potentially differential expression. Compliance with Minimum Information About a Microarray Experiment (MIAME) [22] was assured by depositing all the data in the Gene Expression Omnibus (GEO) repository [23]. The GEO accession numbers are as follows. Platform: GPL9136; Samples: GSM453634, GSM453635, GSM453636, GSM453637 and GSM453638; Series: GSE18145. We confirmed mRNA expression of six picked up SERPIN genes (*SERPINA5, SERPINB6, SERPINE1, SERPINE2, SERPINF2 *and *SERPING1*) using QPCR analysis to validate the results of microarray analysis. All six follicles were used in QPCR analysis. The procedures for QPCR were previously described [24]. The primer sequences for each gene are given in Table 1.

**Table 1:** Details of the primers used for quantitative real-time RT-PCR analysis

Gene name	GeneBank accession number	Primer	Sequences	Position
*SERPINA5*	NM_176646	Forward	5'-TGGAAAATGGCCTGAAGGAA-3'	889-908
		Reverse	5'-ATAAAGCTCAAGCCGCCTCTT-3'	962-942
*SERPINB6*	NM_174789	Forward	5'-AGCACCCAGATTCTGGTTCTTC-3'	725-746
		Reverse	5'-GTGTTCAAGTCCGTGCTTTCAC-3'	810-789
*SERPINE1*	NM_174137	Forward	5'-CAGGCGGACTTCTCCAGTTTT-3'	1097-1117
		Reverse	5'-ACCTCAATCTTCACCTTCTGCAG-3'	1173-1151
*SERPINE2*	NM_174669	Forward	5'-CGTAGCACAGACAGATTTGAAGGA-3'	1068-1091
		Reverse	5'- GCAAAATTCGCCTTTGATGG-3'	1148-1129
*SERPINF2*	NM_174670	Forward	5'-CCTGGAACAATCAGAACAGCTCT-3'	579-601
		Reverse	5'-TAATGTTCGCCAGGTCTTCCC-3'	658-638
*SERPING1*	NM_174821	Forward	5'-GAGATGACCAAGTTCCATCCCA-3'	1081-1102
		Reverse	5'-GTAATCCAGCATGTCCTGACTGC-3'	1158-1136
*GAPDH*	U85042	Forward	5'- ACCCAGAAGACTGTGGATGG-3'	444-463
		Reverse	5'-CAACAGACACGTTGGGAGTG-3'	621-602

### Experiment 2: gene and protein localization of four SERPINs (SERPINA5, SERPINB6, SERPINF2 and SERPING1) in E_2_-active and E_2_-inactive follicles

#### Sample collection and follicular fluid steroid hormone determinations

We obtained eleven large follicles (≥8 mm in diameter) from Japanease Black cows at a local slaughterhouse and aspirated 200 μl of FF from the each follicle for hormone determinations. The follicles were dissected from the ovaries and fixed in 10% formalin, embedded in paraffin wax, and stored at 4°C until *in situ *hybridization and immunohistochemistry. The concentrations of E_2 _and progesterone (P_4_) in the FF samples were determined directly in duplicate using a time-resolved fluorescent immunoassay as described previously [25,26]. We classified follicles into two groups based on the relative levels of E_2 _and P_4 _in FF (E_2_/P_4 _≥1: E_2_-active; E_2_/P_4 _< 1: E_2_-inactive) [15].

#### In situ hybridization

Digoxigenin-labeled anti-sense and sense cRNA probes of each SERPINs were prepared as previously described [27,28]. For hybridization, follicles were sectioned into 7-μm-thick sections. The procedure for *in situ *hybridization using an automated Ventana HX System Discovery with a RiboMapKit and a BlueMapKit (Roche Diagnostics, Basel, Switzerland) was previously described [15,27,28].

#### Immunohistochemistry

Immunohistochemistory was performed using the automated Ventana HX System Discovery with a DabMapKit (Roche) described previously by our laboratory [29]. The 7-μm-thick follicular sections were incubated at room temperature for 4 h with rabbit polyclonal anti-SERPINA5 antibody (H00005104, Abnova, Taipei, Taiwan) diluted 1:10, rabbit polyclonal anti-SERPINB6 antibody (GTX114637, GeneTex Inc, Irvine, CA, USA) diluted 1:100, rabbit polyclonal anti-SERPINF2 antibody (H00005345, Abnova) diluted 1:20 or rabbit polyclonal anti-SERPING1 antibody (GTX105316, GeneTex) diluted 1:300 in Discovery Ab diluents (Roche). The sections were then incubated with anti-rabbit IgG-Biotin conjugate (B7389, Sigma, St. Louis, MO, USA) diluted 1:50 in Discovery Ab diluents (Roche) at room temperature for 1 h. Immunoreactive signals were detected using streptavidin-HRP and diaminobenzidine (DabMapKit, Roche). Counter stain was performed by hematoxylin and bluing reagent (saturated lithium carbonate solution). Antibody specificity of these SERPIN antibodies in bovine follicles was confirmed by Western blot analyses on bovine follicular lysates (GCs and theca interna).

#### Statistical analysis

In experiment 1, the expression ratio of each gene to *GAPDH *mRNA was calculated to adjust for variations in the QPCR reaction. QPCR data in experiment 1 was analyzed by a Mann-Whitney's U test. Results are presented as the mean ± SEM. Statistical significance was considered at *P *< 0.05.

## Results

### Experiment 1

#### Microarray analysis and quantitative real-time PCR analysis

**Figure 1:**
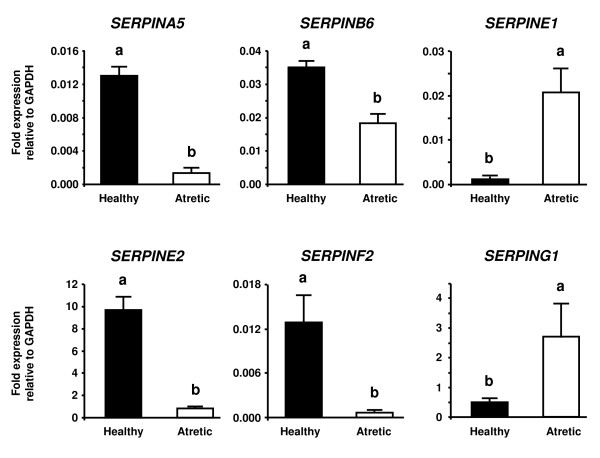
**QPCR analysis of *SERPINA5, SERPINB6, SERPINE1, SERPINE2, SERPINF2 *and *SERPING1 *in healthy and atretic follicles**. Total RNA from the follicular wall (i.e., granulosa plus theca interna) was extracted from three healthy follicles and three atretic follicles. The expression of mRNA was normalized to the expression of *GAPDH *measured in the same RNA preparation. The black and white bars indicate healthy and atretic follicles, respectively. Data are shown as the mean ± SEM. Different superscript letters denote significant differences (*P *< 0.05).

Among 14 genes coding for the SERPIN superfamily represented on a bovine oligonucleotide array, 11 SERPIN genes were identified after data normalization: *SERPINA1, SERPINA5, SERPINB1, SERPINB6, SERPINB8, SERPINE1, SERPINE2, SERPINF1, SERPINF2, SERPING1 *and *SERPINH1*. Of these 11 SERPIN genes, *SERPINA5, SERPINB6, SERPINE2 *and *SERPINF2 *were consistently up-regulated at least 2.0-fold, while *SERPINE1 *and *SERPING1 *were consistently down-regulated at least 0.5-fold in all healthy follicles compared with all atretic follicles. We compared the expression levels of these six SERPINs between healthy and atretic follicles by QPCR. Figure 1 shows the results of QPCR analysis. Consistent with the microarray analysis, *SERPINA5, SERPINB6, SERPINE2 *and *SERPINF2 *mRNA expression was greater in healthy than in atretic follicles (*P *< 0.05). On the other hand, *SERPINE1 *and *SERPING1 *mRNA expression was greater in atretic than in healthy follicles (*P *< 0.05).

### Experiment 2

Among the six SERPINs analyzed by QPCR in experiment 1, we investigated the mRNA and protein localization of four (SERPINA5, SERPINB6, SERPINF2 and SERPING1) in E_2_-active and E_2_-inactive follicles. These SERPINs have not been previously described in bovine ovarian follicles.

### In situ hybridization of four SERPIN genes in E_2_-active and E_2_-inactive follicles

**Figure 2:**
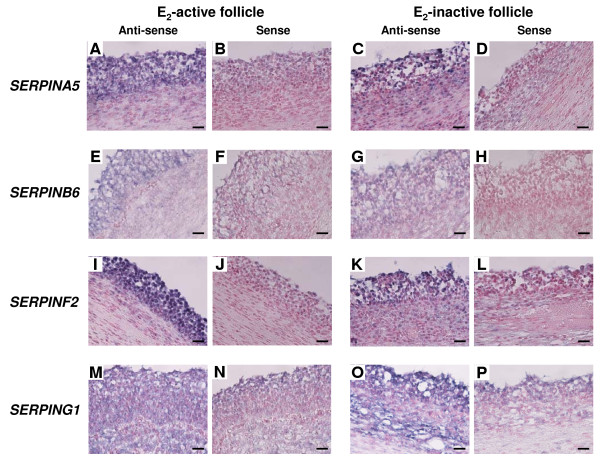
**mRNA localization of *SERPINA5, SERPINB6, SERPINF2 *and *SERPING1 *in E**_**2**_**-active and E**_**2**_**-inactive follicles**. *SERPINA5, SERPINB6 *and *SERPINF2 *mRNA was expressed more in healthy than in atretic follicles, while *SERPING1 *mRNA was expressed more in atretic than in healthy follicles in QPCR analysis. (A, C, E, G, I, K, M and O) Digoxigenin (DIG)-labeled anti-sense cRNA probes were used. (B, D, F, H, J, L, N and P) DIG-labeled sense cRNA probes were used. Sections (7 μm) of bovine follicles were hybridized with each probe. *SERPINA5 *(A, B, C and D), *SERPINB6 *(E, F, G and H) and *SERPINF2 *(I, J, K and L) mRNA were found in the GCs of E_2_-active follicles and a weak hybridization signal was detected in GCs of E_2_-inactive follicles. *SERPING1 *mRNA (M, N, O and P) was detected in both GCs and the TL of E_2_-inactive follicles and a weak hybridization signal was also detected in both GCs and the TL of E_2_-active follicles. Scale bars = 20 μm.

As shown in Figure 2, mRNA expressions of SERPINA5, SERPINB6 and SERPINF2 were detected in GCs of E_2_-active follicles and a weak hybridization signal was also detected in GCs of E_2_-inactive follicles (Figure 2A, C, E, G, I and 2K). *SERPING1 *mRNA was localized in both GCs and the TL of E_2_-inactive follicles and a weak hybridization signal was also detected in both GCs and the TL of E_2_-active follicles (Figure 2M and 2O). No significant signals were detected with any sense probes (Figure 2B, D, F, H, J, L, N and 2P).

### Immunohistochemistry of four SERPIN proteins in E_2_-active and E_2_-inactive follicles

**Figure 3:**
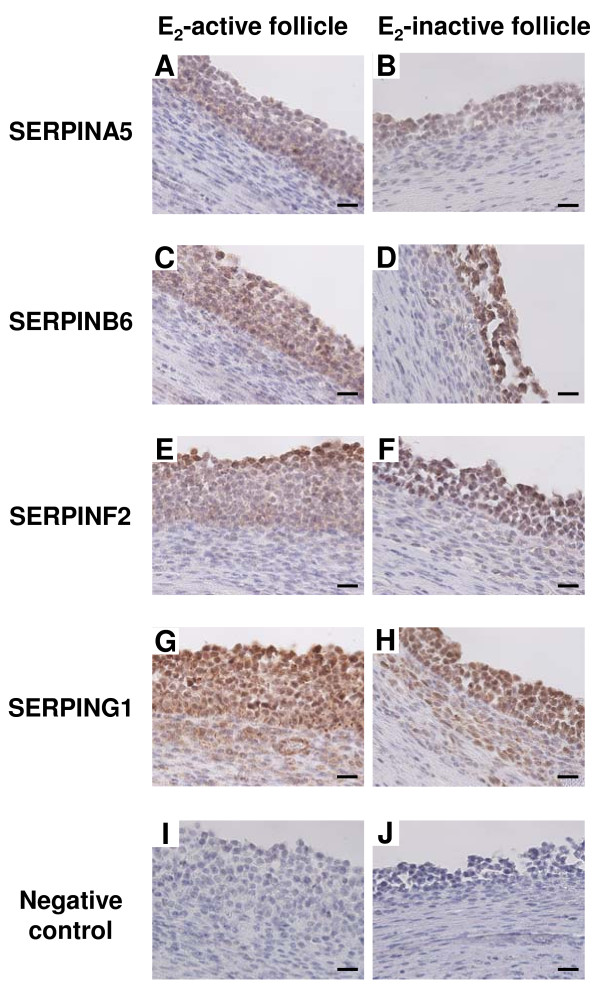
**Protein localization of *SERPINA5, SERPINB6, SERPINF2 *and *SERPING1 *in E**_**2**_**-active and E**_**2**_**-inactive follicles**. Localization of SERPINA5 (A and B), SERPINB6 (C and D), SERPINF2 (E and F) and SERPING1 (G and H) protein was detected by immunohistochemistry. Sections (7 μm) of bovine E_2_-active (A, C, E and G) and E_2_-inactive follicles (B, D, F and H) were incubated with anti-SERPINA5, anti-SERPINB6, anti-SERPINF2 and anti-SERPING1 polyclonal antibodies. SERPINA5, SERPINB6 and SERPINF2 were detected in the GCs of E_2_-active and E_2_-inactive follicles. SERPING1 was detected in both GCs and the TL of E_2_-active and E_2_-inactive follicles. Negative control (I and J) was incubated without anti-SERPIN antibodies. Scale bars = 20 μm.

SERPINA5, SERPINB6 and SERPINF2 were detected in GCs of E_2_-active and E_2_-inactive follicles (Figure 3A, B, C, D, E and 3F). SERPING1 protein was localized in both GCs and the TL of E_2_-active and E_2_-inactive follicles (Figure 3G and 3H).

## Discussion

This study focused on the mRNA expression of SERPINs during bovine follicular development. Microarray and QPCR analyses revealed that a total of 11 SERPINs are expressed in both healthy and atretic follicles. Of these, nine were identified for the first time in bovine follicle while six were differentially expressed between healthy and atretic follicles. We also demonstrate that four of the identified SERPIN genes and proteins showed stage-specific localization in E_2_-active and E_2_-inactive follicles.

In the present study, the mRNA expression of four out of six SERPINs differentially expressed between healthy and atretic follicles was detected for the first time in bovine follicles. These SERPINs are mainly involved in regulation of fibrinolytic, coagulation and protein C pathways. *SERPINA5 *showed higher mRNA expression in healthy than in atretic follicles. This SERPIN is also called protein C inhibitor and regulates the coagulation cascade by following three pathways; 1) inhibition of down-regulation of the coagulation pathway to inhibit activated protein C (APC), 2) anticoagulant function in the presence of heparin by inhibiting proteolytic cleavage of fibrinogen by thrombin, 3) procoagulation function in the presence of thrombomodulin by inhibiting activation of protein C by thrombin [30]. In bovine follicles, GCs express prothorombin (thrombin precursor) and thrombin receptor (PAR-1) mRNA and FF contains prothorombin, heparin sulfate and thrombin-generating proteins (Factor V, VII and X) [31,32], suggesting that the coagulation cascade is locally activated in bovine follicles. Our present result reveals that mRNA and protein of SERPINA5 was localized only in the GCs. Thus, it is plausible that GCs are the main site of action of protein C and thrombin in bovine follicles. In addition, APC has direct anti-inflammatory and anti-apoptotic properties by cleaving PAR-1 [30]. SERPINA5 in healthy follicles may be involved in the regulation of not only the coagulation cascade but also the anti-inflammation and anti-apoptotic functions of APC.

SERPING1 (C1 inhibitor) regulates complement pathway activation and blocks the activity of plasma kallikrein and the activated form of factor XII in blood [33]. These two proteins participate in the production of bradykinin, which promotes inflammation by increasing vascular permeability [34]. The presence of the bradykinin-producing system within follicles has been suggested in porcine since plasma kallikrein and its physiological substrate HMW-kininogen coexist in the FF [35]. We found that *SERPING1 *mRNA was more highly expressed in atretic than in healthy follicles and both mRNA and protein was detected in the GCs and the TL of E_2_-inactive follicles. High expression of *SERPING1 *mRNA in atretic follicles may regulate vascular permeability and suppress inflammation promoted by bradykinin. To support our results, it has been speculated that bradykinin is involved in the selection and atresia of ovarian follicles because the porcine study demonstrated that the concentration of bradykinin in FF of small follicles was higher than in the FF of medium and large follicles [35].

Fibrinolytic cascades, including the PA-plasmin system, play a crucial role in the degradation and remodeling of follicular basal lamina and ECM associated with follicular development, ovulation and atresia [36]. In the present study, both *SERPINE2 *and *SERPINF2 *mRNA were found to be more greatly expressed in healthy than in atretic follicles. SERPINE2 is a potent inhibitor of thrombin, plasmin, both urokinase (uPA) and tissue-type PA (tPA) [37,38]. mRNA and protein expression of SERPINE2 were detected in bovine GCs [19]. In accordance with our results, the FF protein level and mRNA expression of SERPINE2 in GCs were higher in non-atretic than in atretic follicles [21]. SERPINF2 is a known primary physiological inhibitor of plasmin [17,39]. Our results demonstrated for the first time that *SERPINF2 *mRNA is expressed in ovarian follicles and both the mRNA and the protein are localized in the GCs of healthy and atretic follicles. Only GCs express both SERPINE2 and SERPINF2, suggesting that GCs may play an important role in the regulation of the PA-plasmin cascade. Atretic follicles had higher FF plasmin activity than non-atretic follicles while there was no difference in mRNA expression levels of uPA [21]. We previously demonstrated that mRNA expression of uPA receptor was higher in atretic than in healthy follicles [15]. Therefore, we speculate that the follicular PA-plasmin system may mainly be regulated by changes in the balance of expression of their inhibitors and receptors.

Although SERPINE1 as well as SERPINE2 acts as a primary inhibitor of both uPA and tPA [40], its mRNA expression was higher in atretic than in healthy follicles in contrast to *SERPINE2*. A previous study showed that there was no difference in mRNA expression levels of SERPINE1 between non-atretic and atretic bovine follicles [21]. However, *SERPINE1 *mRNA expression may decrease in the process of bovine follicular development since it was down-regulated in large follicles (10 mm in diameter) compared with medium-sized follicles (8 mm in diameter) [14]. SERPINE1 is an acute phase protein and its production dramatically increases in response to cytokines such as interleukin-1β (IL-1β) and tumor necrosis factor α (TNFα) [41,42]. An *in vitro *study of cultured rat GCs revealed that both IL-1β and TNFα suppressed the gonadotropin-induced enhancement of PAs activity, which was accompanied by an increase in plasminogen activator inhibitor activity [43,44]. Expression of *TNFα *mRNA was greater in bovine GCs of subordinate follicles compared with DF [7]. Thus, greater mRNA expression of SERPINE1 in atretic follicles may be stimulated by enhanced ILs and TNFα production during follicular atresia.

Healthy follicles also showed greater expression of *SERPINB6 *mRNA than atretic follicles. In our present study, *in situ *hybridization and immunohistochemistry revealed that mRNA and protein localization of SERPINB6 was restricted only to the GCs of bovine follicles. It has been demonstrated that Spi/SERPINB6, which is the mouse ortholog of SERPINB6, and two Spi3/SERPINB6 paralogs, NK13/SERPINB6b and Spi3C/SERPINB6c, were expressed in a mouse follicular somatic cells and/or oocytes [45]. A primary target protease of SERPINB6 is cathepsin G [46], whereas its gene expression is likely to be restricted to the myeloid lineage [47] while the expression in ovarian follicular cells is unknown. SERPINB6 modulates proteolytic activities of a variety of proteases including plasmin, thrombin, tissue kallikrein and trypsin and β-tryptase [48,49]. mRNA and protein of tissue kallikrein, thrombin and plasmin are localized in GCs of bovine antral follicles and are thought to contribute to regulation of follicular angiogenesis, coagulation or tissue remodeling [32,50]. SERPINB6 is apparently restricted to the cytoplasm of cells and cannot be released via the conventional secretory pathway [51]. Thus, we speculate that SERPINB6 may participate in follicular development to inhibit intracellular proteases in GCs.

Furthermore, we identified for the first time five SERPIN genes (*SERPINA1, SERPINB1, SERPINB8, SERPINF1 *and *SERPINH1*) that are expressed in bovine follicles by microarray analysis. The primary functions of these SERPINs are inhibition of neutrophil elastase (SERPINA1 and SERPINB1) [52,53], inhibition of furin (SERPINB8) [54] and antiangiogenic molecule (SERPINF1) [55]. SERPINH1 is known as heat shock protein 47 and is involved in molecular maturation of collagens to act as a collagen-specific molecular chaperone which does not have a protease inhibitory function [56]. This SERPIN may modulate biosynthesis of collagens in follicles regardless of their health status because the mRNA of collagen types I and IV are detected in GCs and in the basement membrane of bovine follicles [57]. Our results imply that numerous SERPINs may constantly participate in regulation of follicular functions.

## Conclusions

We have identified 11 SERPIN genes that are expressed in bovine follicles by microarray analysis. Of these, six are differentially expressed between healthy and atretic follicles. In addition, we have demonstrated for the first time that mRNA and protein of SERPINA5, SERPINB6 and SERPINF2 showed characteristic localization in the GCs of follicles, whereas mRNA and protein of SERPING1 was localized in both GCs and TL of E_2_-active and E_2_-inactive follicles. Stage-specific expression of SERPINs may participate in the growth and atresia of bovine follicles as important mediators of diverse local mechanisms such as coagulation, protein C and fibrinolytic pathways.

## Competing interests

The authors declare that they have no competing interests.

## Authors' contributions

KGH participated in the design of the study, collected the materials, carried out all experiments and drafted the manuscript. KU collected the materials, carried out the microarray experiments and analysis, and helped to carry out QPCR and *in situ *hybridization. MH was responsible for all animal care, collected the materials and carried out the microarray experiments. TT supervised the study, collected the materials and helped to draft the manuscript. All authors read and approved the final manuscript.
